# Use of targeted therapies for advanced renal cell carcinoma in the Veterans Health Administration

**DOI:** 10.1002/cam4.2531

**Published:** 2019-09-19

**Authors:** Sherrie L. Aspinall, Xinhua Zhao, Mark C. Geraci, Chester B. Good, Francesca E. Cunningham, Bernadette B. Heron, Daniel Becker, Steve Lee, Vinay Prasad, for the Targeted Therapies in Veterans with RCC Study Group, Vida Passero, Jenna Shields, Roslyn A. Stone, Ron Carico, John Szymanski, Alyssa Taqi, Amy Blauvelt, Jennifer Sanderson, Michael Gass, Chelsea Minor, Kourtney LaPlant, Iman Suliman, Elaine Twedt, Megan Nelson, Betsy Paul, Robert Dowd, Sean Keefe, Candice Wenzell, Greg Horn, Brett Carroll, Rob Wenzell, David Panning, Lindsay Kaster, Lindsey Hunt, Katerina Butler, Robert Carr, Camille Kampschmidt, Ann Nawarskas, Ivy Tonnu‐Mihara, Ni‐Chi Wu, Eugene Tseng, Megan Banaszynski, Russell Crawford, Brian Do, Bailey Crandall, Lianna Serbas, Julia Hammond, Kelly Chillari, Marshall Tague, Alison Stauder, Brooke Crawford, Susan Bullington, Jill Mutziger, Joy Meier, Tatiana Sawyer, Janice Taylor, Jason Bena

**Affiliations:** ^1^ VA Pharmacy Benefits Management Services Hines IL; ^2^ VA Center for Health Equity Research and Promotion VA Pittsburgh Healthcare System Pittsburgh PA; ^3^ School of Pharmacy University of Pittsburgh Pittsburgh PA; ^4^ School of Medicine University of Pittsburgh Pittsburgh PA; ^5^ Center for Value‐Based Pharmacy Initiatives UPMC Health Plan Pittsburgh PA; ^6^ New York University School of Medicine New York NY; ^7^ VA NY Harbor Healthcare System New York NY; ^8^ Oregon Health and Science University Portland OR

**Keywords:** renal cell carcinoma, treatment, Veterans

## Abstract

**Background:**

The objective of this study is to describe the use of targeted therapies for the treatment of advanced renal cell carcinoma (RCC) and overall survival (OS) among patients in clinical practice in the Veterans Health Administration (VHA).

**Methods:**

A retrospective cohort of 286 patients from 24 VHA Medical Centers diagnosed with advanced clear cell RCC between Fiscal Year (FY) 2010 and FY2014 was followed through September 30, 2016. Among patients who received targeted therapy, we described the medications taken, duration of therapy, and overall survival. We also assessed the effect of the first therapy received on overall survival using Cox Proportional Hazards models.

**Results:**

There were 66 patients who did not receive therapy for their advanced RCC. Of the 220 treated patients, the mean (sd) number of medications received was 1.9 (1.1). The medications most commonly used first were sunitinib (61.8%), pazopanib (17.3%), and temsirolimus (10.9%). The median duration of first‐line therapy was 86 days (interquartile range [IQR] 42, 210). Median total duration of therapy was 159 days (IQR 58, 397). 62.3% of patients had ≥ 1 dose of therapy held or reduced, mainly due to an adverse drug event (ADE). Median survival from the start of treatment to death was 1.08 years (IQR 0.80, 1.31). Finally, receipt of temsirolimus vs sunitinib (HR 1.95 [95%CI 1.09,3.47]) as the first targeted therapy was independently associated with an increased hazard of death.

**Conclusion:**

Our analysis of targeted therapies for advanced RCC in VHA suggests duration of treatment is shorter in a real‐world setting than in clinical trials, and dose reductions and ADEs are more common.

## Graphical abstract

Our analysis of targeted therapies for advanced renal cell carcinoma in Veterans Health Administration suggests duration of treatment is shorter in a real‐world setting than in clinical trials, and dose reductions and adverse drug events are more common.

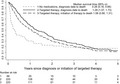

## 1 INTRODUCTION

Despite the approval of multiple targeted therapies for the treatment of metastatic renal cell carcinoma (RCC), including a vascular endothelial growth factor‐antibody (VEGF‐mAb), multitargeted VEGF tyrosine kinase inhibitors, mammalian target of rapamycin inhibitors, and immune checkpoint inhibitors, the optimal sequencing of these agents is unknown, especially beyond second‐line therapy.^1^ Additionally, the degree to which clinical trial results (eg, duration of therapy, overall survival) match real‐world outcomes remains uncertain.^2^

The 21st Century Cures Act was signed into law in December of 2016.^3^ Among its features is the creation of a framework allowing for the use of real‐world data or real‐world evidence (RWE) to support regulatory decision making. RWE is defined by the FDA as, “data regarding the usage, or the potential benefits or risks, of a drug derived from sources other than traditional clinical trials.” The use of RWE can overcome some of the restraints seen in randomized control trials, such as patients with few comorbidities, idealized conditions based on exclusion criteria, inadequate assessment of risk, and lack of generalizability. However, RWE may support incorrect causal inference, may utilize large data sets of uncertain quality, and requires appropriate analytic methods.^4^, ^5^, ^6^, ^7^, ^8^

The Veterans Health Administration (VHA) is the largest integrated health care system in the United States. The quality of care provided in VHA is equivalent to, or exceeds, the quality seen in the private sector for both cancer^9^, ^10^ and noncancer care.^11^, ^12^ The objective of this study was to describe the use of sequential targeted therapies for the treatment of advanced RCC and overall survival (OS) among patients in real‐world clinical practice in VHA.

## 2 PATIENTS AND METHODS

### 2.1 Patients

This was a retrospective cohort study of patients diagnosed with advanced clear cell RCC between Fiscal Year (FY) 2010 and FY2014 and treated at 1 of 24 VHA medical centers across the country; patients were followed through September 30, 2016. Local cancer registries were used to identify patients with a diagnosis of stage IV or initial recurrence of kidney cancer; then, VHA electronic medical records (ie, Computerized Patient Record System or CPRS) were reviewed to ascertain the subset of patients with pathology‐confirmed clear cell, or predominantly clear cell, RCC. If the local cancer registry was known to be incomplete (eg, registrar position vacant for a period), then additional methods (eg, data warehouse) were used to identify patients. Patients who received treatment for RCC outside of VHA were excluded, unless they received their initial prescription for ≤30 days of a single medication from a non‐VHA provider, then transferred the remainder of their care to VHA. In addition, patients who received their diagnosis at VHA, then transferred their care elsewhere, were excluded. Those who left to participate in a clinical trial were censored. Finally, we excluded a small number of patients who received treatment with both targeted and nontargeted medications. These exclusions were applied because our goal was to examine targeted therapies prescribed in VHA. The Institutional Review Boards for participating sites and VA Pharmacy Benefits Management Services approved the study.

### 2.2 Data collection

For patients with RCC, pharmacists reviewed CPRS and the cancer registry to record the date of diagnosis, type of surgery and/or ablative therapy for this episode (eg, nephrectomy, radiation), history of other cancers, and Eastern Cooperative Oncology Group (ECOG) performance status prior to initiation of any targeted therapy. Comorbidities, demographics, and smoking status were obtained from the VA Medical SAS Datasets (Austin Information Technology Center in Austin, TX).

In those patients who were treated for RCC, pharmacists used CPRS to collect data on targeted therapies (ie, axitinib, cabozantinib, pazopanib, sorafenib, sunitinib, bevacizumab, everolimus, temsirolimus, nivolumab) received so we could determine the sequence of medications and total duration of treatment. In addition, reasons a medication was discontinued or a dose was reduced/held were recorded. Any adverse drug events (ADEs) were recorded and graded according to the National Cancer Institute Common Terminology Criteria for Adverse Events (CTCAE) version 4.0. For those who were not treated with medications, the reasons were documented. Finally, dates of death were obtained from the Vital Status file.

### 2.3 Statistical analysis

Baseline characteristics of patients, including demographics, smoking status, comorbidities as defined in the Deyo et al adaptation of the Charlson Comorbidity Index,^13^ ECOG performance status, year of diagnosis, type of surgery/ablative therapy, and geographic region of the VA medical center were described overall and by treatment status (ie, receipt of targeted therapy, no medication, or nontargeted plus targeted therapy). Chi‐square or Fisher exact tests were used to compare categorical variables between the two groups, and Wilcoxon tests were used to compare continuous variables.

For descriptive purposes, we explained the main reasons why patients were not treated with any medications for their advanced RCC. Among patients who received targeted therapy, we described the number of medications received, the sequencing of medications (eg, 1st, 2nd), and duration of therapy, both by place in therapy and medication. We also described the proportion of patients who had one or more doses of at least one targeted therapy held or reduced, overall and by medication, and the rationale. Similar descriptive analyses were conducted for patients who discontinued targeted therapies.

Kaplan‐Meier survival curves were used to summarize OS from diagnosis date to death by receipt of targeted therapy vs no medication. An “immortal time” bias created by the waiting time prior to initiation of treatment, and the sample size was small for untreated patients.^14^, ^15^ Therefore, we focused on those who received targeted therapy as planned, with survival time defined as time since the initiation of therapy to death or end of follow‐up. An OS curve, without the immortal waiting time, was added to the Kaplan‐Meier curves. We assessed median waiting time from date of diagnosis until initiation of targeted therapy. We also calculated median OS from initiation of therapy until death and by first targeted therapy received. Finally, we examined the effect of the first targeted therapy received on OS using a Cox Proportional Hazards model with robust standard errors clustered at the site level to account for grouping of patients within site. Variables, including patient baseline characteristics and time from diagnosis to the start of targeted therapy, associated with OS at *P* < .20 in the bivariate analyses were included in the multivariate model. Age and race/ethnicity were forced into the model. To account for missing data in key covariates (race for 8.6%, smoking for 4.1%, and ECOG score for 38.2% of observations), we used multiple imputation by chained equations across 10 multiply imputed datasets.^16^ Unadjusted and adjusted hazard ratios (HRs) and 95% CIs were reported.

Given the large proportion of patients missing a baseline ECOG score, we assessed whether baseline characteristics differed by missing vs nonmissing ECOG scores, and then, we ran a sensitivity analysis of the multivariable Cox model using dummy variables for missing values instead of multiple imputation to assess the robustness of the model results. We also assessed the baseline characteristics for the eight patients who received both nontargeted and targeted therapy and were excluded from the analyses. Analyses were performed using SAS version 9.4 (SAS Institute Inc, Cary, NC) and STATA 14 (College Station, TX).

## 3 RESULTS

### 3.1 Patient cohort and baseline characteristics

**Figure 1:**
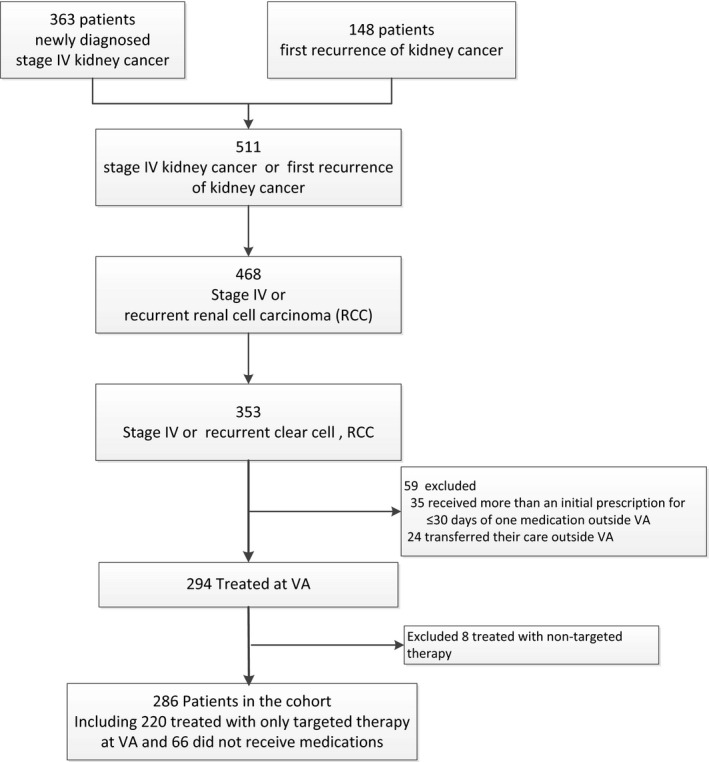
Construction of cohort with advanced renal cell carcinoma

Of the 511 patients who presented with stage IV kidney cancer or an initial recurrence of their kidney cancer, 353 had pathology‐confirmed clear cell, RCC (Figure 1). After excluding patients who did not receive care at VHA (n = 59) or were treated with both nontargeted and targeted therapy (n = 8), 286 patients remained in the cohort.

The mean age of patients with advanced RCC was 66.3 years (Table 1). All were male, and 70.6% were white. Baseline characteristics of patients who received targeted therapy and those who received no medications for advanced RCC were similar except for ECOG performance status; a greater percentage of patients who received treatment were in the ECOG categories of 0, 1, and 2. A high percentage in both groups had an unknown performance status.

**Table 1:** Baseline characteristics of patients with advanced renal cell carcinoma by treatment status

	Total (N = 286) n (col %)	Received targeted therapy (N = 220, 76.9%) n (col %)	No therapy with medications (N = 66, 23.1%) n (col %)	*P*‐value^a^
Age (years), mean (SD)	66.3 (8.6)	65.8 (8.5)	67.7 (8.6)	.11
<65	132 (46.2)	106 (48.2)	26 (39.4)	.45
65‐74	112 (39.2)	83 (37.7)	29 (43.9)	
>=75	42 (14.7)	31 (14.1)	11 (16.7)	
Male	286 (100.0)	220 (100.0)	66 (100.0)	/
Race/ethnicity				.64
White	202 (70.6)	159 (72.3)	43 (65.2)	
Black	27 (9.4)	18 (8.2)	9 (13.6)	
Hispanic	24 (8.4)	17 (7.7)	7 (10.6)	
Other	9 (3.1)	7 (3.2)	2 (3.0)	
Unknown	24 (8.4)	19 (8.6)	5 (7.6)	
Married	136 (47.6)	109 (49.5)	27 (40.9)	.22
Smoking status				.21
No	185 (64.7)	139 (63.2)	46 (69.7)	
Yes	92 (32.2)	72 (32.7)	20 (30.3)	
Unknown	9 (3.1)	9 (4.1)	0 (0.0)	
Charlson Comorbidity Index, mean (sd)	5.5 (3.7)	5.4 (3.8)	6.0 (3.5)	.23
Charlson Comorbidity Index, median (IQR)	5.0 (2.0, 9.0)	5.0 (2.0, 9.0)	6.0 (3.0, 9.0)	.23
Positive Cancer History (other than kidney)	57 (19.9)	40 (18.2)	17 (25.8)	.18
Year of Diagnosis (advanced RCC)				.96
FY2010	32 (11.2)	25 (11.4)	7 (10.6)	
FY2011	70 (24.5)	56 (25.5)	14 (21.2)	
FY2012	69 (24.1)	52 (23.6)	17 (25.8)	
FY2013	72 (25.2)	54 (24.5)	18 (27.3)	
FY2014	43 (15.0)	33 (15.0)	10 (15.2)	
Type of Surgery or Ablative Therapy				
Any surgery or ablative therapy	121 (42.3)	92 (41.8)	29 (43.9)	.76
Partial nephrectomy	7 (2.4)	5 (2.3)	2 (3.0)	.73
Radical nephrectomy	80 (28.0)	61 (27.7)	19 (28.8)	.87
Ablative techniques or radiation therapy	18 (6.3)	13 (5.9)	5 (7.6)	.62
Surgical metastasectomy	21 (7.3)	16 (7.3)	5 (7.6)	.93
Other	3 (1.0)	3 (1.4)	0 (0.0)	.43
ECOG Performance Status				.01
0	34 (11.9)	28 (12.7)	6 (9.1)	
1	75 (26.2)	64 (29.1)	11 (16.7)	
2	37 (12.9)	33 (15.0)	4 (6.1)	
3	13 (4.5)	8 (3.6)	5 (7.6)	
4	6 (2.1)	3 (1.4)	3 (4.5)	
Unknown	121 (42.3)	84 (38.2)	37 (56.1)	
Advanced RCC Diagnosis Type				.46
Stage IV on presentation	202 (70.6)	153 (69.5)	49 (74.2)	
Initial recurrence of RCC	84 (29.4)	67 (30.5)	17 (25.8)	
Region of VA Medical Center				.81
Midwest	99 (34.6)	74 (33.6)	25 (37.9)	
West	95 (33.2)	74 (33.6)	21 (31.8)	
South	53 (18.5)	40 (18.2)	13 (19.7)	
Northeast	39 (13.6)	32 (14.6)	7 (10.6)	

Abbreviations: ECOG, Eastern Cooperative Oncology Group; RCC, renal cell carcinoma.

a *P*‐value for “targeted therapy” vs “no therapy with medications” for advanced RCC.

Patients with an ECOG score vs those missing a baseline score had similar characteristics except for advanced RCC diagnosis type. Patients missing an ECOG score were more likely to be classified as having an initial recurrence vs presenting with stage IV disease (39% vs 25%) (Table S1). The baseline characteristics of the eight patients who got nontargeted and targeted therapy were comparable with both patients who received and did not receive medications for their advanced RCC, but their ECOG scores were all 0 (62.5%), 1 (25.0%), or 2 (12.5%) (Data not shown).

### 3.2 Patients who did not receive treatment

Of the 286 patients in the cohort, 66 (23.1%) did not receive any medications for their advanced RCC; the main reasons were poor performance status (34.9%), extent of disease (33.3%), patient refusal (22.7%), comorbidities (18.2%), and physician opinion that the patient would not tolerate therapy (18.2%). Patients could have more than one reason. The median duration of follow‐up (date of diagnosis until death or the end of the study period) for patients who did not receive medications for RCC was 0.29 years (interquartile range or IQR 0.11, 1.52).

### 3.3 Patients who received targeted therapy

Among those 220 patients who were treated with only targeted therapy, the median duration of follow‐up was 1.30 years (IQR 0.58, 2.64). During this time, the mean (SD) number of medications received was 1.9(1.1). Almost half (49.1%) received a second targeted therapy, 25.9% received a third, and 10.5% received a fourth (Table 2). A total of 14 patients had five or six lines of targeted therapy. The therapies most frequently used first were sunitinib (61.8%), pazopanib (17.3%), and temsirolimus (10.9%). Those used second included everolimus (35.2%), sorafenib (16.7%), axitinib (14.8%), and pazopanib (13.9%), and the targeted therapies frequently used third were everolimus (33.3%), axitinib (21.1%), and sorafenib (19.3%).

**Table 2:** Targeted therapy used to treat advanced renal cell carcinoma and duration of therapy

	Overall n (col%)	Place in therapy‐first n (col%)	Place in therapy‐second n (col%)	Place in therapy‐third n (col%)	Place in therapy‐fourth^a^ n (col%)	Duration of therapy, *by Medication* Median days (IQR)
Overall (n, % of those who received targeted therapy)	220	220 (100.0)	108 (49.1)	57 (25.9)	23 (10.5)	159 (58, 397)
Targeted Therapy						
Axitinib	37 (16.8)	2 (0.9)	16 (14.8)	12 (21.1)	4 (17.4)	59 (30, 102)
Cabozantinib^b^	2 (0.9)	1 (0.5)	0	0	0	
Pazopanib	65 (29.5)	38 (17.3)	15 (13.9)	6 (10.5)	2 (8.7)	135 (65, 240)
Sorafenib	43 (19.5)	8 (3.6)	18 (16.7)	11 (19.3)	3 (13.0)	63 (29, 150)
Sunitinib	155 (70.5)	136 (61.8)	10 (9.3)	7 (12.3)	2 (8.7)	86 (42, 246)^c^
Everolimus	71 (32.3)	10 (4.5)	38 (35.2)	19 (33.3)	4 (17.4)	84 (47, 168)
Temsirolimus	33 (15)	24 (10.9)	7 (6.5)	0	1 (4.3)	56 (28, 84)^c^
Nivolumab	13 (5.9)	1 (0.5)	3 (2.8)	2 (3.5)	6 (26.1)	49 (28, 70)^c^
Bevacizumab^b^	2 (0.9)	0	0	0	1 (4.3)	
Duration of therapy, *by Place in Therapy* Median days (IQR)		86 (42, 210)	75 (32,150)	88 (32,152)	60 (42,126)	

Abbreviation: IQR, interquartile range.

a Fourteen patients had ≥ five lines of therapy, with a maximum of six lines for two patients.

b Duration of therapy not shown for two patients who received cabozantinib and bevacizumab.

c For sunitinib, once daily ×4 weeks, then 2 wk off, repeat, was counted as 42 d on medication. Similar calculations were done for other medications that are not dosed every day.

The median total duration of targeted therapy was 159 days (ie, 5.2 months) (Table 2). The median duration of therapy was similar by place in therapy for the medications used first (86 days), second (75 days), and third (88 days), but a drop‐off occurred with the fourth medication (60 days). Nivolumab had the shortest median duration of therapy (49 days), and pazopanib had the longest median duration of therapy (135 days). When duration of therapy was assessed by accounting for both medication and place in therapy, the median duration decreased for most targeted therapies if they were used later during treatment (eg, sunitinib as first medication‐median duration of 110 days [interquartile range or IQR 42, 252], sunitinib as second medication‐74 days [IQR 32, 105], sunitinib third‐63 days [IQR 32, 210]) (Table S2). However, the median duration of therapy increased for pazopanib when it was used later during treatment, and sorafenib showed no trend (Table S2).

### 3.4 Alteration and discontinuation of targeted therapy

Of the 220 patients who received targeted therapy, 137 (62.3%) had one or more doses of at least one targeted therapy held or reduced; the percentages of patients ranged from 31.0% for everolimus to 54.5% for temsirolimus. For sorafenib, axitinib, pazopanib, and sunitinib, the percentages were 37.2%, 43.2%, 47.7%, and 51.0%, respectively. The most common reason for holding or reducing a dose was an ADE (83.9%).

In addition, 89.1% of patients discontinued one or more targeted therapies during the study period. The percentages ranged from 70.3% for axitinib to 94.4% for everolimus, with sorafenib (76.7%), sunitinib (84.5%), pazopanib (90.8%), and temsirolimus (90.9%) falling in between. Disease progression was the most common reason (59.7%), followed by ADEs (44.9%). Of the ADEs that led to discontinuation of the targeted therapy, the most frequent events were thrombocytopenia, nausea/vomiting/diarrhea, hand‐foot skin reaction, hepatotoxicity, acute kidney injury/renal failure, other rash, and pneumonitis. In addition, 22.7% (20/88) of the ADEs were severe (ie, grades 3‐5).

### 3.5 Overall survival and survival by first targeted therapy received

**Figure 2:**
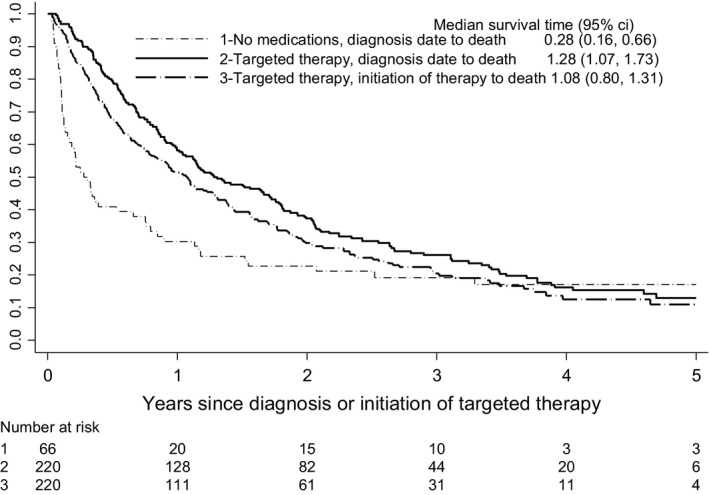
Overall survival in patients receiving targeted therapy (from date of diagnosis and from initiation of therapy) and in patients receiving no medication

Of the 220 patients who received targeted therapy, 81.4% died by the end of the study period. Median overall survival from date of diagnosis was 1.28 years (IQR 1.07, 1.73) (Figure 2). The 1‐ and 3‐year median survival rates were 0.58 (IQR 0.51, 0.64) and 0.26 (IQR 0.20, 0.32), respectively (ie, 58% and 26% of patients who received targeted therapy were alive 1 year and 3 years after diagnosis, respectively). However, the median time (“immortal waiting time”) from diagnosis to the start of targeted therapy was 0.13 years (IQR 0.10, 0.15), and median survival from the start of treatment to death was 1.08 years (IQR 0.80, 1.31) (Figure 2). In those patients who received no medications for their advanced RCC, 83.3% (55/66) died by the end of the study; median survival was 0.28 years (IQR 0.16, 0.66) from the date of diagnosis to death (Figure 2). For the eight patients who received both nontargeted and targeted therapy, 50% died by the end of the study period. Median survival from the start of treatment until death was 4.06 years (IQR 0.34, ‐).

**Figure 3:**
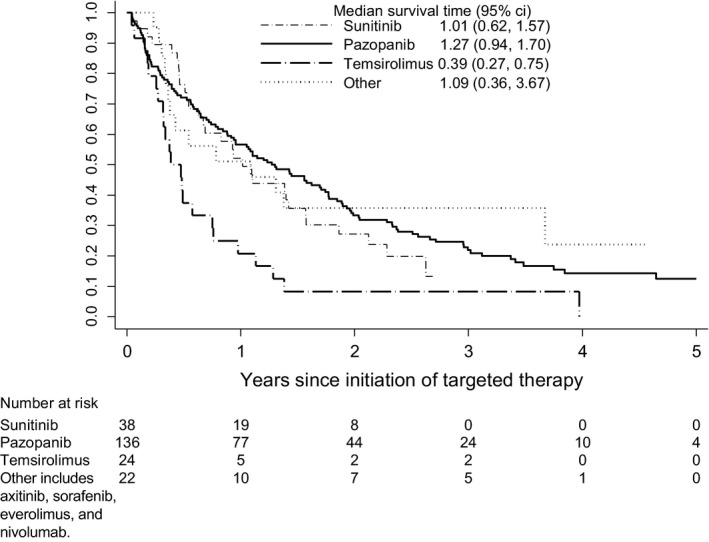
Overall survival by first targeted therapy received

Among the targeted therapies most commonly used first, patients who received temsirolimus had the shortest median survival from the start of therapy to death (0.39 years [IQR 0.27, 0.75]) (Figure 3). Those who received pazopanib first had the longest median survival (1.27 years [IQR 0.94, 1.70]), and sunitinib fell in between with a median survival of 1.01 years (IQR 0.62, 1.57).

### 3.6 Effect of first targeted therapy on overall survival

After adjusting for other factors that could influence survival, receipt of temsirolimus compared with sunitinib (HR 1.95 [95%CI 1.09,3.47]) as the first targeted therapy was associated with an increased hazard of death (Table 3), and undergoing any type of surgery or ablative therapy vs none (HR 0.69 [95%CI 0.49,0.97]) was associated with a decreased hazard of death (Table 3). In addition, use of targeted therapy for an initial recurrence of RCC vs presenting with stage IV disease (HR 0.53 [95%CI 0.35,0.80]) was associated with a decreased risk of death (Table 3).

**Table 3:** Effect of first‐line targeted therapy on overall survival^a^

	Unadjusted model		Adjusted model	
	HR (95% CI)	*P*‐value	aHR (95% CI)	*P*‐value
First‐line therapy				
Sunitinib	Reference		Reference	
Pazopanib	1.19 (0.82,1.71)	.37	1.21 (0.82,1.79)	.33
Temsirolimus	2.23 (1.32,3.78)	.003	1.95 (1.09,3.47)	.02
Other^b^	0.86 (0.48,1.56)	.62	0.93 (0.52,1.67)	.82
Age				
<65	Reference		Reference	
65‐74	0.88 (0.64,1.21)	.43	0.83 (0.59,1.16)	.27
≥75	0.98 (0.68,1.42)	.94	1.09 (0.72,1.66)	.67
Race/Ethnicity				
White	Reference		Reference	
Black	0.90 (0.45,1.81)	.77	0.77 (0.34,1.70)	.51
Hispanic	0.82 (0.34,1.98)	.66	0.62 (0.23,1.62)	.33
Other	0.56 (0.17,1.87)	.34	0.41 (0.11,1.54)	.19
Married	0.75 (0.61,0.92)	.006	0.84 (0.63,1.11)	.22
Smoking	1.32 (1.01,1.72)	.04	1.36 (0.92,1.99)	.12
Charlson comorbidity index	1.04 (1.00,1.08)	.04	1.02 (0.97,1.06)	.49
Any surgery or ablative therapy	0.68 (0.49,0.94)	.021	0.69 (0.49,0.97)	.03
ECOG performance status				
0	Reference		Reference	
1‐2	1.28 (0.89,1.86)	.19	1.43 (0.96,2.14)	.08
3‐4	1.26 (0.64,2.50)	.50	1.23 (0.63,2.41)	.54
Advanced RCC diagnosis type				
Stage IV on presentation	Reference		Reference	
Initial recurrence of RCC	0.56 (0.39, 0.81)	.002	0.53 (0.35,0.80)	.003

Abbreviations: ECOG, Eastern Cooperative Oncology Group; RCC, renal cell carcinoma.

a Survival time was from start of targeted therapy to death or end of follow‐up. Variables, including all patient baseline characteristics and time from diagnosis to the start of targeted therapy, associated with overall survival at *P* < .20 from bivariate analysis were included in the multivariable model. Age and race/ethnicity were forced into the model. We used multiple imputation (20 imputations) for ethnicity/race (8.6% missing), smoking (4.1% missing), and ECOG score (38.2% missing) using chained equations.

b Other includes axitinib, sorafenib, everolimus, and nivolumab.

The sensitivity analysis that used dummy variables for missing values instead of multiple imputation yielded similar results (<15% change in point estimates) (Table S3).

## 4 DISCUSSION

Our report from 24 VHA medical centers across the country complements and expands upon other retrospective analyses of real‐world utilization of renal cell carcinoma therapies.^17^, ^18^, ^19^, ^20^, ^21^, ^22^, ^23^ To our knowledge, this analysis is the first to quantify the gains in overall survival that are likely attributable to guarantee or immortal time (about 44 days). Guarantee time refers to the period that a patient must live prior to the initiation of anticancer therapy to be included in the treatment group in retrospective analyses.^24^

There are external observations that strengthen the validity of our findings. First, we find diminished survival with temsirolimus as the initial therapy when compared to other agents. Given temsirolimus is commonly used in patients with poor performance status, based on randomized trials testing the drug in those with at least three predictors of shortened survival,^25^ this observation is expected. We find a general similarity in overall survival between initial therapy with sunitinib and pazopanib, which also mirrors the results of a randomized trial by Motzer and colleagues.^26^

There are also several notable findings in our study that differ from published clinical trials. First, we observed a median duration of initial therapy of 86 days or just under 3 months. This is lower than the median duration of treatment of 8.0 and 7.6 months for pazopanib and sunitinib, respectively, in a large randomized trial.^27^ Second, median survival from diagnosis in our cohort of those receiving at least one targeted therapy was 1.28 years; this was 2.36 years and 2.43 years for pazopanib and sunitinib, respectively, in the trial by Motzer et al.^26^ There is growing recognition that the explicit inclusion criteria and implicit selection biases of randomized trials may result in patient populations that are younger and have fewer comorbidities than the average cancer patient.^28^ Even if the efficacy of anticancer drugs is preserved on a relative basis (ie, the hazard ratio of benefit remains), lower baseline survival means a lower absolute benefit that may affect the benefit/harm ratio of cancer drugs. Patients should be counseled about these differences. Third, dose reductions and discontinuations were common in our cohort. We found that 62.3% of patients had one or more doses of targeted therapy held or reduced. ADEs were a common reason for dose reductions and the discontinuation of therapy. Of concern, we found that 22.7% of ADEs that led to discontinuation of the medication were grade 3‐5. These results should be interpreted alongside prior studies showing that access to and quality of care at VHA is, on average, the same or significantly better than non‐VA hospitals.^9^, ^10^, ^11^, ^12^ As such, our results raise the question of whether, or to what degree, clinical trial evidence applies to average patient populations.

In contrast with prior observational studies,^17^, ^18^, ^19^, ^20^, ^21^, ^22^, ^23^ we provide an estimate of immortal time. Figure 2 shows the difference in overall survival between patients who receive no therapy and patients who receive at least one targeted therapy. There are three factors that account for the difference between the two groups of patients: (a) patient prognostic characteristics that determined whether treatment was given; (b) the benefit provided by therapy; and (c) guarantee time (prior to the receipt of medication) in the group that ultimately received at least one drug. By providing two Kaplan‐Meier curves, one from diagnosis and the other from the start of therapy, we show the potential contribution of guarantee time. The remainder of the difference is attributable to the former two factors. This is important because overall survival may be overstated in observational studies if this bias is not considered.^17^, ^20^ For instance, in an analysis of the effect of targeted therapy on overall survival in mRCC using the Surveillance, Epidemiology, and End Results (SEER) cancer registry, the authors found a median OS of 20 months; the index date is not stated, but appears to be the date of diagnosis.^20^ This is longer than what we found, even when starting with the diagnosis date, and likely reflects differences in the patient populations.

To avoid this guarantee bias, observational studies have used an index date corresponding to the start of therapy when determining overall survival.^21^, ^22^, ^23^ For example, a retrospective analysis from the International Metastatic Renal Cell Carcinoma Database Consortium (IMDC) described the use of mammalian target of rapamycin (mTOR) inhibitors as first‐line therapy for mRCC and found that patients who received temsirolimus had a shorter median OS than those who got everolimus (12.5 vs 15.9 months from the start of therapy until death, respectively).^22^ The authors of another study using the IMDC found no difference in median OS between sunitinib and pazopanib (22.3 vs 22.6 months, respectively).^23^ Again, OS was shorter in our Veteran population, but the conclusions are similar.

### 4.1 Limitations

Although our work is illustrative of patterns of utilization of anticancer drugs for advanced RCC and provides insight into their tolerability and efficacy, there are several limitations. Our work is subject to all the limitations inherent to retrospective studies even though we secured data from a representative set of VHA medical centers. It is not well suited for cross drug comparisons and cannot be used to conclude the superiority or inferiority of one targeted therapy vs another. Also, our population was male, so results may be different in females; although, other factors are probably more clinically relevant drivers of our findings. In addition, our results predominantly describe those who were treated for advanced RCC, and they appear to be “healthier” than untreated patients. Finally, we were unable to collect the data needed to calculate the IMDC prognostic risk category, which may have influenced the treatment decision and overall survival. However, we did include the ECOG Performance Status, which provides some insight regarding the patient's prognosis.

## 5 CONCLUSION

Our retrospective analysis of targeted therapy for advanced RCC in VHA suggests the median duration of treatment is shorter in real‐world use than what is observed in the clinical trials that guide practice, with frequent ADEs, dose reductions, and discontinuations. Collectively, these factors likely erode the benefits of these agents in the real world. The clinical trials agenda in RCC warrants reassessment to better align the information generated to that of patients who receive these medications in practice, patients who generally carry a worse prognosis and tolerate medication for shorter periods of time. Patients and physicians should be cognizant of, and discuss, these issues and the potential impact on survival.

## DISCLOSURES

Dr. Prasad reports receiving: royalties from his book, *Ending Medical* *Reversal*; funding from the Laura and John Arnold Foundation, and honoraria for Grand Rounds/lectures from several universities, medical centers, nonprofit groups, and professional societies. He is a writer for Medscape and runs the podcast “Plenary Session,” which has Patreon backers. The views expressed in this paper are those of the authors, and no official endorsement by the Department of Veterans Affairs or the United States Government is intended or should be inferred.

## AUTHORS’ CONTRIBUTIONS

The authors meet the ICMJE definition of authorship. Dr. Aspinall was involved in conceptualization, formal analysis, methodology, and writing—original draft. Dr. Zhao was involved in conceptualization, data curation, formal analysis, methodology, and writing—original draft. Dr. Geraci and Dr. Prasad were involved in conceptualization, methodology, and writing—original draft. Dr. Good, Dr. Cunningham, Dr. Heron, and Dr. Becker were involved in conceptualization, methodology, and writing—review and editing. 

## APPENDIX 1

### Members of the Targeted Therapies in Veterans with RCC Study Group

Vida Passero, MD^1^; Jenna Shields, PharmD^1^; Roslyn A. Stone, PhD^1^; Ron Carico, PharmD, MPH^1^; John Szymanski, PharmD^2^; Alyssa Taqi, PharmD^2^; Amy Blauvelt, PharmD^3^; Jennifer Sanderson, PharmD^4^; Michael Gass, PharmD^5^; Chelsea Minor, PharmD^5^; Kourtney LaPlant, PharmD^6^; Iman Suliman, PharmD^6^; Elaine Twedt, PharmD, MS^7^; Megan Nelson, PharmD^8^; Betsy Paul, PharmD^9^; Robert Dowd, PharmD^9^; Sean Keefe, PharmD^10^; Candice Wenzell, PharmD^11^; Greg Horn, PharmD^11^; Brett Carroll, PharmD^11^; Rob Wenzell, PharmD^11^; David Panning, PharmD^11^; Lindsay Kaster, PharmD^12^; Lindsey Hunt, PharmD^12^; Katerina Butler, PharmD^13^; Robert Carr, PharmD^14^; Camille Kampschmidt, PharmD^14^; Ann Nawarskas, PharmD^14^; Ivy Tonnu‐Mihara, PharmD^15^; Ni‐Chi Wu, PharmD^15^; Eugene Tseng, PharmD^15^; Megan Banaszynski, PharmD^16^; Russell Crawford, BSPharm^16^; Brian Do, PharmD^16^; Bailey Crandall, PharmD^17^; Lianna Serbas, PharmD^17^; Julia Hammond, PharmD^18^; Kelly Chillari, PharmD^18^; Marshall Tague, PharmD^19^; Alison Stauder, PharmD^20^; Brooke Crawford, PharmD^21^; Susan Bullington, PharmD^21^; Jill Mutziger, PharmD^21^; Joy Meier, PharmD^22^; Tatiana Sawyer, PharmD^22^; Janice Taylor, PharmD^23^; and Jason Bena, PharmD^23^

^1^VA Pittsburgh Healthcare System, ^2^VA Connecticut Healthcare System, ^3^Syracuse VA Medical Center, ^4^Robley Rex VA Medical Center, ^5^South Texas Veterans Health Care System, ^6^North Florida/South Georgia Veterans Health System, ^7^William Jennings Bryan Dorn VA Medical Center, ^8^Minneapolis VA Health Care System, ^9^VA Ann Arbor Healthcare System, ^10^Kansas City VA Medical Center, ^11^Louis Stokes Cleveland VA Medical Center, ^12^Boise VA Medical Center, ^13^George E. Wahlen Department of VA Medical Center, ^14^New Mexico VA Health Care System, ^15^VA Long Beach Healthcare System, ^16^Southern Arizona VA Healthcare System, ^17^VA San Diego Healthcare System, ^18^Durham VA Medical Center, ^19^Iowa City VA Medical Center, ^20^John Cochran VA Medical Center, ^21^Richard L. Roudebush VA Medical Center, ^22^VA Northern California Health Care System, and ^23^VA Sierra Nevada Health Care System
