# Supplementary material for: Use of targeted therapies for advanced renal cell carcinoma in the Veterans Health Administration
Source: Cancer Med. 2019 Sep 19;8(15):6651–61. doi: 10.1002/cam4.2531 (PMC6825975; doi:10.1002/cam4.2531)
Supplement: Supplementary file 1 [file CAM4-8-6651-s001.docx]

**eTable 1. Baseline Characteristics of Patients with Advanced Renal Cell Carcinoma by Non-Missing Versus Missing ECOG Scores**

|  | **Total**  (N=220)  n (col %) | **ECOG Non-missing**  (N=136, 61.8%)  n (col %) | **ECOG Missing**  (N=84, 38.2%)  n (col %) | **P-value** |
| --- | --- | --- | --- | --- |
| **Age** (years), mean (sd) | 65.8 (8.5) | 65.8 (7.7) | 65.9 (9.8) | 0.77 |
| <65 | 106 (48.2) | 62 (45.6) | 44 (52.4) | 0.25 |
| 65-74 | 83 (37.7) | 57 (41.9) | 26 (31.0) |  |
| >=75 | 31 (14.1) | 17 (12.5) | 14 (16.7) |  |
| **Male** | 220 (100.0) | 136 (100.0) | 84 (100.0) |  |
| **Race/ethnicity** |  |  |  | 0.38 |
| White | 17 (7.7) | 13 (9.6) | 4 (4.8) |  |
| Black | 159 (72.3) | 97 (71.3) | 62 (73.8) |  |
| Hispanic | 18 (8.2) | 13 (9.6) | 5 (6.0) |  |
| Other | 7 (3.2) | 3 (2.2) | 4 (4.8) |  |
| Unknown | 19 (8.6) | 10 (7.4) | 9 (10.7) |  |
| **Married** | 109 (49.5) | 62 (45.6) | 47 (56.0) | 0.14 |
| **Smoking Status** |  |  |  | 0.53 |
| No | 139 (63.2) | 86 (63.2) | 53 (63.1) |  |
| Yes | 72 (32.7) | 46 (33.8) | 26 (31.0) |  |
| Unknown | 9 (4.1) | 4 (2.9) | 5 (6.0) |  |
| **Charlson Comorbidity Index**, mean (sd) | 5.4 (3.8) | 5.5 (3.9) | 5.1 (3.6) | 0.40 |
| **Positive Cancer History** (other than kidney) | 40 (18.2) | 27 (19.9) | 13 (15.5) | 0.41 |
| **Year of Diagnosis** (advanced RCC) |  |  |  | 0.96 |
| FY2010 | 25 (11.4) | 16 (11.8) | 9 (10.7) |  |
| FY2011 | 56 (25.5) | 36 (26.5) | 20 (23.8) |  |
| FY2012 | 52 (23.6) | 30 (22.1) | 22 (26.2) |  |
| FY2013 | 54 (24.5) | 34 (25.0) | 20 (23.8) |  |
| FY2014 | 33 (15.0) | 20 (14.7) | 13 (15.5) |  |
| **Type of Surgery or Ablative Therapy** |  |  |  | 0.61 |
| None | 128 (58.2) | 77 (56.6) | 51 (60.7) |  |
| Partial or radical nephrectomy | 66 (30.0) | 44 (32.4) | 22 (26.2) |  |
| Other | 26 (11.8) | 15 (11.0) | 11 (13.1) |  |
| **ECOG Performance Status** |  |  |  | / |
| 0 | 28 (12.7) | 28 (20.6) | 0 (0.0) |  |
| 1 | 64 (29.1) | 64 (47.1) | 0 (0.0) |  |
| 2 | 33 (15.0) | 33 (24.3) | 0 (0.0) |  |
| 3 | 8 (3.6) | 8 (5.9) | 0 (0.0) |  |
| 4 | 3 (1.4) | 3 (2.2) | 0 (0.0) |  |
| Unknown | 84 (38.2) | 0 (0.0) | 84 (100.0) |  |
| **Advanced RCC Diagnosis Type** |  |  |  | 0.03 |
| Stage IV on presentation | 153 (69.5) | 102 (75.0) | 51 (60.7) |  |
| Initial recurrence of RCC | 67 (30.5) | 34 (25.0) | 33 (39.3) |  |

**eTable 2. Duration of Targeted Therapy by Place in Therapy and Medication**^†^

|  | **Overall**  Median days  (IQR) | **Place in Therapy-**  **First**  Median days  (IQR) | **Place in Therapy-Second**  Median days  (IQR) | **Place in Therapy-**  **Third**  Median days  (IQR) |
| --- | --- | --- | --- | --- |
| **Overall**  Median days (IQR) | 159 (58, 397) | 86 (42, 210) | 75 (32,150) | 88 (32,152) |
| **Targeted Therapy** |  |  |  |  |
| Axitinib | 59 (30, 102) | / | 53 (20, 98) | 44 (22, 107) |
| Pazopanib | 135 (65, 240) | 95 (55, 160) | 150 (120, 190) | 441 (340, 616) |
| Sorafenib | 63 (29, 150) | 64 (28, 227) | 56 (21, 240) | 90 (41, 120) |
| Sunitinib^‡^ | 86 (42, 246) | 110 (42, 252) | 74 (32, 105) | 63 (32, 210) |
| Everolimus | 84 (47, 168) | 97 (42, 120) | 84 (50, 168) | 63 (30, 150) |
| Temsirolimus | 56 (28, 84) | 67 (28, 85) | 42 (14, 49) | / |

IQR=interquartile range

^†^Duration of therapy was only shown for cells with sample size of 4 or above. Cabozantinib, nivolumab, bevacizumab, and place in therapy beyond third medication were not listed in the table because the cell sizes are too small.

^‡^For sunitinib, once daily x4 weeks, then 2 weeks off, repeat, was counted as 42 days on medication. Similar calculations were done for other medications that are not dosed every day.

**eTable 3**. **Effect of First-line Targeted Therapy on Overall Survival using Dummy Variables for Missing Values^†^**

|  | **Unadjusted Model** | | **Adjusted Model** | |
| --- | --- | --- | --- | --- |
|  | **HR (95% CI)** | **P-value** | **aHR (95% CI)** | **P-value** |
| **First-line Therapy** |  |  |  |  |
| Sunitinib | reference |  | reference |  |
| Pazopanib | 1.19 (0.82,1.71) | 0.37 | 1.21 (0.83,1.77) | 0.32 |
| Temsirolimus | 2.23 (1.32,3.78) | 0.003 | 2.27 (1.28,4.01) | 0.005 |
| Other | 0.86 (0.48,1.56) | 0.62 | 0.99 (0.55,1.79) | 0.98 |
| **Age** |  |  |  |  |
| <65 | reference |  | reference |  |
| 65-74 | 0.88 (0.64,1.21) | 0.43 | 0.89 (0.62,1.26) | 0.50 |
| ≥75 | 0.98 (0.68,1.42) | 0.94 | 1.15 (0.74,1.79) | 0.53 |
| **Race/Ethnicity** |  |  |  |  |
| White | reference |  | reference |  |
| Black | 0.91 (0.44,1.89) | 0.80 | 0.82 (0.45,1.51) | 0.53 |
| Hispanic | 0.83 (0.34,2.03) | 0.69 | 1.51 (0.66,3.46) | 0.33 |
| Other/missing | 0.48 (0.15,1.60) | 0.23 | 0.87 (0.45,1.67) | 0.66 |
| **Married** | 0.75 (0.61,0.92) | 0.006 | 0.81 (0.61,1.08) | 0.15 |
| **Smoking Status** |  |  |  |  |
| No | reference |  | reference |  |
| Yes | 1.35 (1.03,1.77) | 0.03 | 1.37 (0.93,2.02) | 0.11 |
| Unknown | 1.18 (0.83,1.68) | 0.35 | 1.13 (0.57,2.24) | 0.72 |
| **Charlson Comorbidity Index**, mean (sd) | 1.04 (1.00,1.08) | 0.04 | 1.02 (0.97,1.07) | 0.45 |
| **Any Surgery or Ablative Therapy** | 0.68 (0.49,0.94) | 0.02 | 0.73 (0.52,1.03) | 0.07 |
| **ECOG Performance Status** |  |  |  |  |
| 0 | reference |  | reference |  |
| 1-2 | 1.56 (0.97,2.49) | 0.07 | 1.68 (1.08,2.62) | 0.02 |
| 3-4 | 1.26 (0.51,3.13) | 0.61 | 1.19 (0.56,2.53) | 0.65 |
| Unknown | 1.82 (1.03,3.22) | 0.04 | 2.26 (1.37,3.73) | 0.001 |
| **Advanced RCC Diagnosis Type** |  |  |  |  |
| Stage IV on presentation | reference |  | reference |  |
| Initial recurrence of RCC | 0.56 (0.39,0.81) | 0.002 | 0.51 (0.34,0.78) | 0.002 |

ECOG=Eastern Cooperative Oncology Group; RCC=renal cell carcinoma

^†^Survival time was from start of targeted therapy to death or end of follow up. Variables, including all patient baseline characteristics and time from diagnosis to the start of targeted therapy, associated with overall survival at P<0.20 from bivariate analysis were included in the multivariable model. Age and race/ethnicity were forced into the model. We created separate dummy variables for missing values of ethnicity/race (8.6% missing), smoking (4.1% missing), and ECOG score (38.2% missing) instead of multiple imputation.
